# NMR elucidation of nonproductive binding sites of lignin models with carbohydrate-binding module of cellobiohydrolase I

**DOI:** 10.1186/s13068-020-01805-w

**Published:** 2020-10-07

**Authors:** Yuki Tokunaga, Takashi Nagata, Keiko Kondo, Masato Katahira, Takashi Watanabe

**Affiliations:** 1 Research Institute for Sustainable Humanosphere (RISH), Kyoto University, Uji, Kyoto 611-0011 Japan; 2 Institute of Advanced Energy (IAE), Kyoto University, Uji, Kyoto 611-0011 Japan

**Keywords:** Cellulase, Lignin, NMR, Carbohydrate-binding module, Cellobiohydrolase I, Enzyme adsorption, Chemical shift perturbation, HSQC, Lignin oligomer model

## Abstract

**Background:**

Highly efficient enzymatic saccharification of pretreated lignocellulose is a key step in achieving lignocellulosic biorefinery. Cellobiohydrolase I (Cel7A) secreted by *Trichoderma reesei* is an industrially used cellulase that possesses carbohydrate-binding module 1 (*Tr*CBM1) at the C-terminal domain. The nonproductive binding of *Tr*CBM1 to lignin significantly decreases the enzymatic saccharification efficiency and increases the cost of biomass conversion because of the additionally required enzymes. Understanding the interaction mechanism between lignin and *Tr*CBM1 is essential for realizing a cost-effective biofuel production; however, the binding sites in lignin have not been clearly elucidated.

**Results:**

Three types of ^13^C-labeled β-*O*-4 lignin oligomer models were synthesized and characterized. The 2D ^1^H–^13^C heteronuclear single-quantum correlation (HSQC) spectra of the ^13^C-labeled lignin models confirmed that the three types of the ^13^C labels were correctly incorporated in the (1) aromatic rings and β positions, (2) α positions, and (3) methoxy groups, respectively. The *Tr*CBM1-binding sites in lignin were analyzed by observing NMR chemical shift perturbations (CSPs) using the synthetic ^13^C-labeled β-*O*-4 lignin oligomer models. Obvious CSPs were observed in signals from the aromatic regions in oligomers bound to *Tr*CBM1, whereas perturbations in the signals from aliphatic regions and methoxy groups were insignificant. These findings indicated that hydrophobic interactions and π–π stacking were dominating factors in nonproductive binding. The synthetic lignin models have two configurations whose terminal units were differently aligned and donated C^(I)^ and C^(II)^. The C^(I)^ ring showed remarkable perturbation compared with the C^(II)^, which indicated that the binding of *Tr*CBM1 was markedly affected by the configuration of the lignin models. The long-chain lignin models (degree of polymerization (DP) 4.16–4.70) clearly bound to *Tr*CBM1. The interactions of *Tr*CBM1 with the short-chain lignin models (DP 2.64–3.12) were insignificant, indicating that a DP greater than 4 was necessary for *Tr*CBM1 binding.

**Conclusion:**

The CSP analysis using ^13^C-labeled β-*O*-4 lignin oligomer models enabled the identification of the *Tr*CBM1 binding sites in lignins at the atomic level. This specific interaction analysis will provide insights for new molecular designs of cellulase having a controlled affinity to cellulose and lignin for a cost-effective biorefinery process.

## Background

**Fig. 1:**
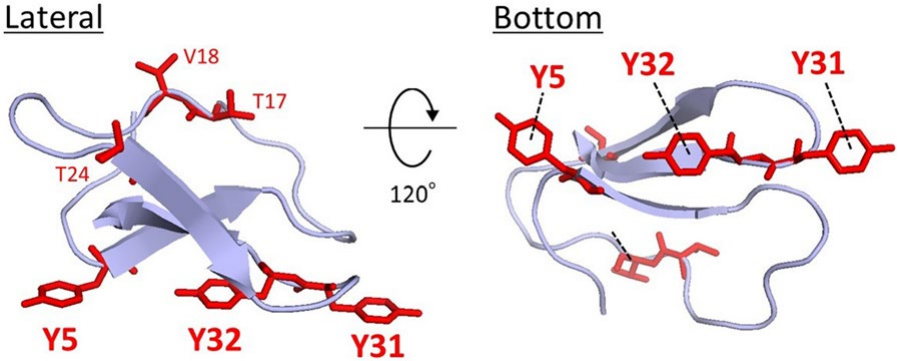
Ribbon models of *Tr*CBM1 viewed from the side and bottom. The structure of *Tr*CBM1 was determined using the NMR analysis (PDB ID 2CBH) [2]. Red stick models of tyrosine residues (Y5, Y31, and Y32) and T17, V18, and T24 residues in the cleft are also displayed

The enzymatic saccharification of lignocellulose is a key process for the sustainable manufacture of green chemicals and biofuels [1]. *Trichoderma reesei* is a filamentous fungus widely used for producing commercially available cellulolytic enzyme cocktails. Cellobiohydrolase I (Cel7A) accounts for up to 60% of the cellulase secreted by *T. reesei*, and carbohydrate-binding module 1 (*Tr*CBM1) (Fig. 1) is connected to the C-terminal of the Cel7A catalytic domain by a highly glycosylated linker [3]. *Tr*CBM1 enhances enzymatic activity by binding to cellulose [4, 5]. *Tr*CBM1 also has a strong affinity for lignin; hence, lignin significantly inhibits the enzymatic saccharification of pretreated lignocellulose [6, 7]. Thus, the nonproductive binding of *Tr*CBM1 to lignin should be suppressed to achieve efficient enzymatic saccharification. However, the interaction mechanism is not entirely understood at the molecular level.

The binding of *Tr*CBM1 to lignin is affected by various saccharification conditions, such as temperature [8], pH [9, 10], and substrate concentration [11, 12]. The chemical properties of lignin critically influence its cellulase-binding affinity. Many pretreatments increase the number of phenolic OH groups in lignin and its degree of condensation, which enhance the binding between cellulase and lignin [13, 14]. Electrostatic repulsion attributed to large numbers of aliphatic OH groups and negatively charged functionalities, such as carboxyl and sulfone groups, interferes with the cellulase binding [13, 15]. Softwood lignin is mainly synthesized by the radical coupling of guaiacyl monomers, whereas hardwood lignin is synthesized from both guaiacyl (G) and syringyl (S) monomers. The ratio of syringyl to guaiacyl units, denoted by S/G, is an important factor that governs the physicochemical properties of lignified plant cell walls. The S/G ratio affects the nonproductive binding, but it is not the decisive factor for adsorption. Guo et al. found that a low S/G ratio corresponded to a high adsorption capacity [16]. Yang et al. reported that organosolv lignin isolated from softwood lodgepole pine exhibited a higher adsorption affinity for commercial cellulase than lignin from hardwood poplar [17]. However, genetic engineering studies have suggested the opposite, and comparisons of enzymatic hydrolysis in alfalfa cultivars and *Eucalyptus* mutants with high and low S/G ratios have yielded inconsistent results [18, 19]. Although the relationship between the chemical structure of lignin and its binding affinity for cellulolytic enzymes and CBMs has been extensively researched, the evidence for identifying the binding atoms in whole lignin molecules is entirely lacking. This is because no effective method for identifying the interactive sites in lignin, a heterogeneous polymer, is available.

The NMR chemical shift perturbation (CSP) analysis is a powerful method for identifying substrate-binding sites in proteins [20, 21]. We previously performed CSP analysis to identify the binding amino acid residues in *Tr*CBM1 with softwood and hardwood milled wood lignin (MWL) using ^15^N-labeled *Tr*CBM1 [22]. The results suggested that the aromatic rings in the lignin participated in interactions with the amino acid residues in *Tr*CBM1 because the flat plane surface, including Y5, Y31, and Y32 in *Tr*CBM1, was the main interaction site. However, the binding sites in lignin have not been characterized at the molecular level.

Lignin is a heterogeneous polymer comprising C6 − C3 phenylpropane units. These units are connected via different interunit bonding patterns; however, β-*O*-4 aryl ether is the dominant interunit linkage. Therefore, β-*O*-4 lignin oligomer models have been used to investigate the physicochemical properties and (bio)chemical reactivity of lignin [23, 24]. Recently, to analyze the conformational changes in the core lignin structure in solvents with different water contents, we synthesized lignin oligomer models composed of guaiacyl units interconnected solely via β-*O*-4 bonds with the *erythro* configuration [25]. In the present study, we focus on the analysis of the molecular interaction between *Tr*CBM1 and the guaiacyl lignin model whose NMR signals were completely assigned [25]. To perform the CSP analysis of the binding sites with high sensitivity and distinctiveness, we labeled the β-*O*-4 lignin oligomer model compounds with ^13^C at different positions.

To monitor perturbations in the signals obtained from the binding sites in the model compounds, pure *Tr*CBM1 without a catalytic domain and linker was expressed and purified and then added to NMR sample tubes containing the ^13^C-labeled lignin models. The NMR analysis of the ^13^C-labeled lignin model compounds provided the first direct evidence for identifying the binding atoms in the linear lignin chains. The results agreed well with our previous binding site analysis of the protein counterpart, *Tr*CBM1 with MWL [22]. Herein, we provide direct evidence to reveal the interaction sites in the β-*O*-4 lignin substructure binding to *Tr*CBM1.

## Results

### Expression and purification of *Tr*CBM1

*Escherichia coli* BL21 (DE3) was used to express a histidine (His) tag–*Tr*CBM1–green fluorescent protein (GFP) fusion protein. To obtain *Tr*CBM1, the His tag and GFP regions were removed by adding enterokinase and thrombin, respectively. In our previous research, to analyze the molecular mass and conformation of ^15^N-labeled *Tr*CBM1, we performed sodium dodecyl sulfate-polyacrylamide gel electrophoresis (SDS-PAGE), matrix-assisted laser desorption/ionization time-of-flight mass spectrometry (MALDI-TOF-MS), and 2D ^1^H–^15^N heteronuclear single-quantum correlation (HSQC) NMR [22]. In this work, SDS-PAGE and MALDI-TOF-MS were performed to characterize unlabeled *Tr*CBM1. Pure *Tr*CBM1 is a single protein with a molecular mass of 5195.8 Da. The MALDI-TOF-MS spectrum of *Tr*CBM1 and a full-length SDS-PAGE gel are shown in Figure S1 of the Additional file 1.

### Synthesis and characterization of the lignin models

**Fig. 2:**
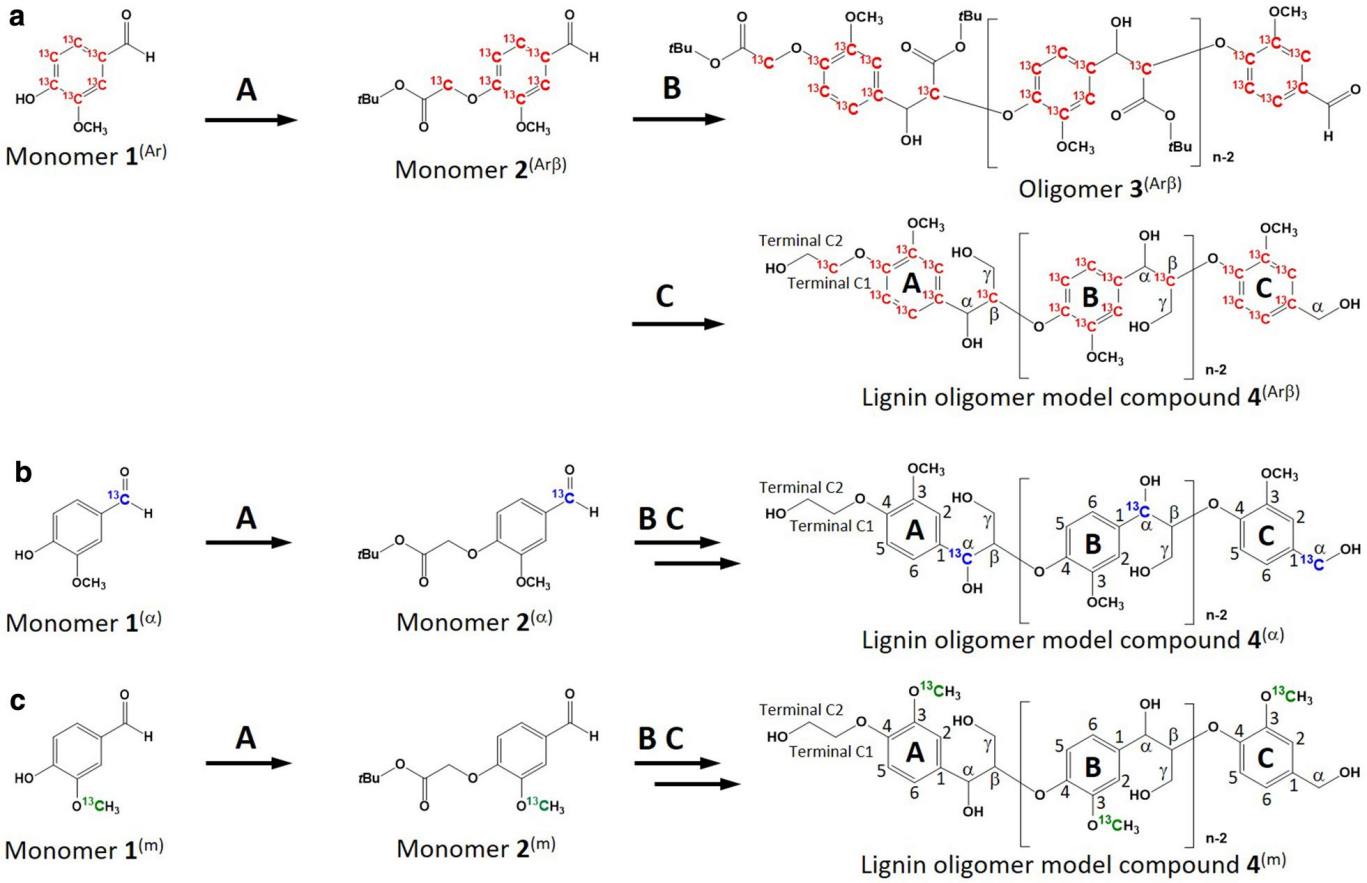
Synthesis of ^13^C-labeled β-*O*-4 lignin oligomer model compounds. ^13^C-labels in **a** aromatic rings and the β positions; **b** α positions; and **c** methoxy groups. A: Vanillin (**1**) was refluxed with BrCH_2_COO*t*Bu, KI, and K_2_CO_3_ in acetone for 1.5 h. B:** 2** is dissolved in anhydrous THF, followed by LDA addition with stirring at −30 °C (1 h). The mixture was then stirred at 0 °C–5 °C for 1.5 h. C:** 3** is dissolved in *t*BuOH with NaBH_4_, followed by the addition of MeOH and stirring at 65 °C–70 °C for 7 h

Recently, we synthesized unlabeled β-*O*-4 lignin oligomer model compound **4** (Fig. 2); its NMR signals were completely assigned to all carbon atoms and nonexchangeable protons by conducting 1D ^1^H NMR, 1D ^13^C NMR, 2D ^1^H–^13^C HSQC, 2D ^1^H–^13^C heteronuclear multiple bond correlation (HMBC), and 2D ^1^H–^13^C long-range heteronuclear single-quantum multiple bond correlation (LR-HSQMBC) experiments [25]. We used the protocol to synthesize three ^13^C-labeled β-*O*-4 lignin oligomer model compounds. The ^13^C labels were placed in the aromatic rings and β positions of compound **4**^(Arβ)^, α positions of compound **4**^(α)^, and methoxy groups of compound **4**^(m)^ using ^13^C-labeled vanillins and *t*-butyl-2-bromoacetate. The ^13^C-vanillins **1**^(Arβ)^, **1**^(α)^, and **1**^(m)^ were individually refluxed in acetone with unlabeled or ^13^C-labeled *t*-butyl-2-bromoacetate to afford ^13^C-*t*-butoxycarbonylmethyl vanillins **2**^(Arβ)^, **2**^(α)^, and **2**^(m)^, respectively. The ^13^C-*t*-butoxycarbonylmethyl vanillins were dissolved in anhydrous tetrahydrofuran (THF) and polymerized using lithium diisopropylamide (LDA). The ester groups in polymerized ^13^C-oligomers **3**^(Arβ)^, **3**^(α)^, and **3**^(m)^ were reduced using NaBH_4_ to obtain ^13^C-labeled β-*O*-4 lignin oligomer model compounds **4**^(Arβ)^, **4**^(α)^, and **4**^(m)^, respectively.

**Fig. 3:**
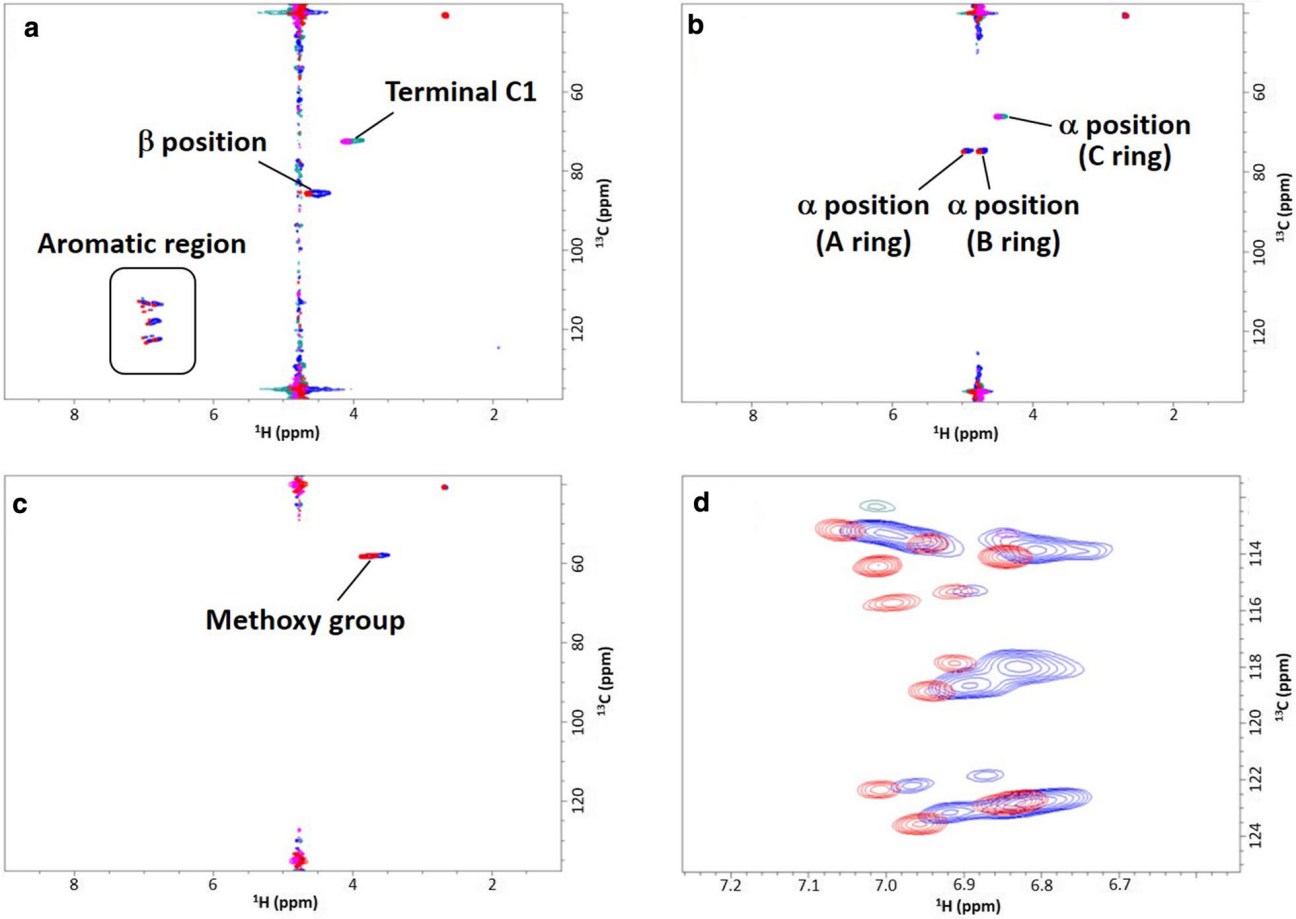
2D ^1^H–^13^C HSQC spectra of ^13^C-labeled β-*O*-4 lignin oligomer model compounds. Superimposed 2D ^1^H–^13^C HSQC spectra of the long- and short-chain models in 50 mM acetic acid-*d*_4_ buffer prepared using D_2_O (pD 5.0) and 10% (v/v) DMSO-*d*_6_ are shown in blue and red, respectively. 2D ^1^H–^13^C HSQC spectra of the lignin oligomer models with **a** ^13^C-labeled aromatic rings and β positions (**4**^(Arβ)^); **b** ^13^C-labeled α positions (**4**^(α)^); and **c** ^13^C-labeled methoxy groups (**4**^(m)^). **d** Magnified view of the aromatic region in (**a**). The noise observed in ^1^H-chemical shift of 4.75 corresponded to the solvent residual peak of water

Figure 3 shows the 2D ^1^H–^13^C HSQC spectra of the ^13^C-labeled lignin models. Recently, we reported the HSQC spectra of the unlabeled lignin model [25]. A comparison of the HSQC spectra of the ^13^C-labeled and unlabeled lignin models indicated that the HSQC signal intensity was significantly enhanced by the incorporation of ^13^C, unequivocally demonstrating that ^13^C was incorporated into the designated positions in the labeled model. No HSQC signals attributable to the side products were observed in the NMR spectra, suggesting the fabrication of high-purity lignin models. The HSQC signals at δ_C_/δ_H_ 85.9/4.52–4.64, 72.6/4.06–4.08, and 113.2–123.6/6.81–7.06 ppm in the HSQC spectra of lignin model **4**^(Arβ)^ with ^13^C labeling in the β positions and aromatic rings (Fig. 3a) were assigned to the β positions, Terminal C1, and aromatic regions, respectively. The HSQC spectra of lignin model **4**^(α)^ (Fig. 3b) contained three signals at δ_C_/δ_H_ 74.7–75.0/4.92–4.98, 74.8–75.0/4.72–4.76, and 66.2/4.43–4.49 ppm, corresponding to the α positions in the A, B, and C rings, respectively. A single signal was observed at 58.1–58.3/3.57–3.85 ppm in the HSQC spectra of lignin model **4**^(m)^ with ^13^C-labeled methoxy groups (Fig. 3c).

The long- and short-chain lignin models were separated via silica gel chromatography by sequential elution with (1) ethyl acetate, (2) ethyl acetate/methanol (5:1, v/v), and (3) ethyl acetate/methanol (2:1, v/v) for NMR and binding analyses. Size exclusion chromatography (SEC) revealed that the ^13^C-labeled lignin models exhibited narrow molecular weight distributions (Additional file 1: Figure S2). The degree of polymerization (DP) was calculated for each model compound based on its weight-average molecular weight (*M*w). The DPs of the long- and short-chain lignin models ranged from 4.16 to 4.70 and from 2.64 to 3.12, respectively (Table 1). Although differences between the *M*ws of the long- and short-chain models were small, their NMR spectra (Fig. 3d) and SEC profiles (Additional file 1: Figure S2) clearly differed. Therefore, the long- and short-chain lignin models could be used to evaluate the effects of *M*w on molecular interactions with *Tr*CBM1.

**Table 1:** Molecular weight parameters of ^13^C-labeled and unlabeled β-*O*-4 lignin oligomer model compounds

Lignin models	Chain length	*M*_n_	*M*_w_	*M*_w_/*M*_n_	DP^a^
Compound **4**^(Arβ)^ (^13^C-labeled aromatic rings and β positions)	Long	843	923	1.095	4.70
	Short	480	520	1.083	2.64
Compound **4**^(α)^ (^13^C-labeled α positions)	Long	750	918	1.224	4.67
	Short	531	614	1.156	3.12
Compound **4**^(m)^ (^13^C-labeled methoxy groups)	Long	629	818	1.301	4.16
	Short	482	587	1.218	2.98
Compound **4** (unlabeled)	Long	882	964	1.092	4.91
	Short	552	560	1.014	2.85

^a^*DP* Degree of polymerization calculated from the *M*w and theoretical molecular mass of the lignin model

### Analysis of interactions between lignin models and *Tr*CBM1

The interactions between the lignin models and *Tr*CBM1 were evaluated via CSP analysis using 2D ^1^H–^13^C HSQC NMR. To observe the NMR signal perturbation with high clarity, the concentration of *Tr*CBM1 should be several times higher than that of the lignin model. However, owing to experimental limitations for preparing a large amount of high-purity *Tr*CBM1, we used the ^13^C-labeled lignin model to decrease the concentration of the additive. The lignin models are composed of two terminal units (A and C rings) and internal units (B rings), which are solely interlinked via the β-*O*-4 linkages with the *erythro* configuration. In the synthesized lignin model, only one alignment of the A and B rings was found, although the C ring exhibited two different alignments designated as C^(I)^ and C^(II)^ [25]. In 90% D_2_O, both configurations adopted the folded conformation; however, C^(II)^ had a slightly more compact conformation than C^(I)^ [25].

**Fig. 4:**
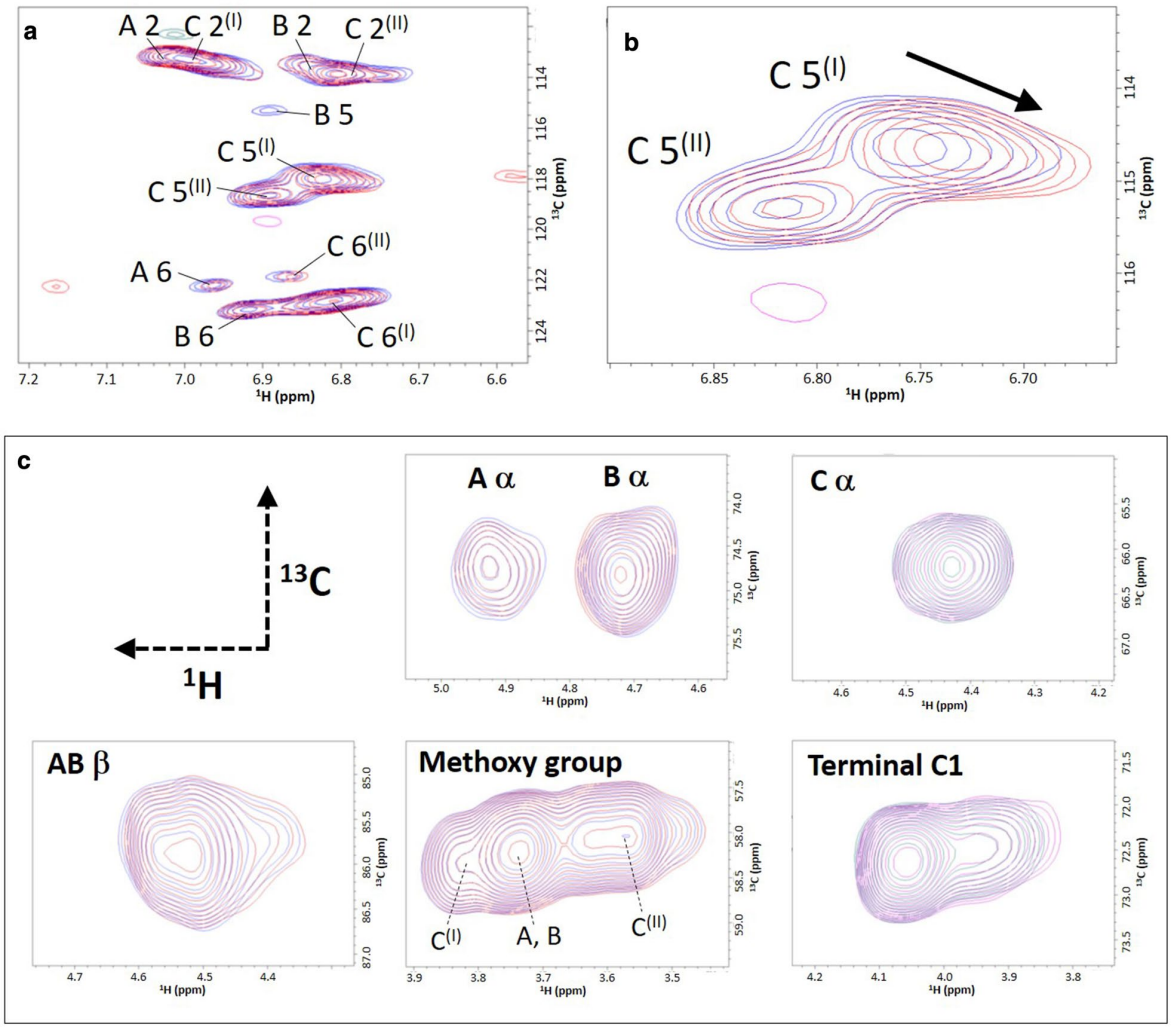
2D ^1^H–^13^C HSQC spectra of the long-chain ^13^C-labeled lignin oligomer models in the CSP experiment. **a** Superimposed 2D ^1^H–^13^C HSQC spectra of the aromatic region in compound** 4**^(Arβ)^ (50 μM) in the presence (red) and absence (blue) of 350 μM *Tr*CBM1. **b** A magnified image of the HSQC signals from the C 5 positions. **c** Superimposed 2D ^1^H–^13^C HSQC spectra of aliphatic regions and methoxy groups in** 4**^(Arβ)^,** 4**^(α)^, and** 4**^(m)^ (50 μM) in the presence (red) and absence (blue) of 350 μM *Tr*CBM1

The 2D ^1^H–^13^C HSQC spectra of the lignin models (50 μM) in the presence and absence of *Tr*CBM1 were superimposed (Fig. 4). Several HSQC signals acquired in the presence of 200 μM *Tr*CBM1 showed obvious perturbation, particularly those in the aromatic regions in the long-chain lignin spectra. Most of these signals exhibited larger perturbations when the concentration of *Tr*CBM1 was increased to 350 μM. The observed perturbations in the aromatic ring are shown in a close-up view (Fig. 4b), and these clear perturbations are good indicators of the interaction [26]. Conversely, the NMR signals obtained from the aliphatic regions and methoxy groups in the presence of *Tr*CBM1 showed no significant perturbation (Fig. 4c). These results clearly indicated that the aromatic regions in the long-chain lignin models were the primary sites for interactions with *Tr*CBM1. However, no distinct perturbations were observed in the signals acquired from the methoxy groups or any of the aromatic and aliphatic regions when the short-chain lignin models were used for CSP analysis (Additional file 1: Figures S3). This indicated that the length of the lignin chains was an important factor in *Tr*CBM1 binding.

**Fig. 5:**
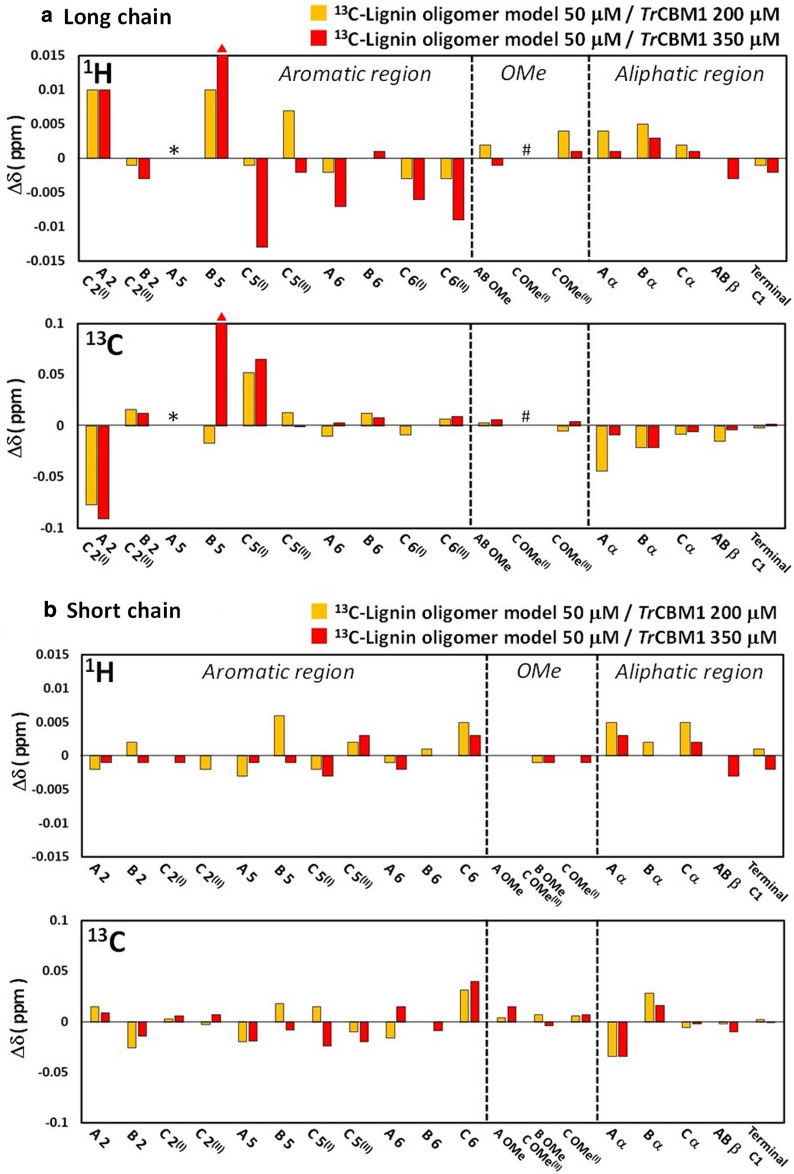
Changes in the chemical shift (Δδ) observed in the CSP analysis. The CSP analysis of the ^13^C-labeled lignin oligomer models (50 μM) was performed in the presence of 200 and 350 μM of *Tr*CBM1 in 50 mM acetic acid-*d*_4_ buffer prepared using D_2_O (pD 5.0) and 10% (v/v) DMSO-*d*_6_. The Δδ values of signals from the **a** long- and **b** short-chain lignin oligomer models in the CSP experiment. The upper panels in **a** and **b** show Δδ on the ^1^H axis. The lower panels show Δδ on the ^13^C axis. Line broadening of the NMR signals from B 5 in the long-chain lignin models was observed in the presence of 350 μM *Tr*CBM1. Positions that generated extremely low-intensity signals in the absence of *Tr*CBM1 are indicated by asterisks (*). Positions that generated overlapping signals are indicated by # symbols

The change in the chemical shift (Δδ) of each signal was calculated by subtracting the chemical shift value in the spectrum of the lignin model from that of the model recorded in the presence of *Tr*CBM1. The ^13^C–^13^C coupling was not eliminated in the HSQC spectra. The CSP analysis was performed by tracing the center of the ^13^C–^13^C coupling signals with high accuracy because each signal was distinct in the HSQC spectra. The Δδ values obtained on the ^1^H and ^13^C axes are plotted in Fig. 5. The Δδ values were large only for the signals obtained from the aromatic regions in the long-chain lignin models, whereas those were small for the signals acquired from the aliphatic regions and methoxy groups in both the long- and short-chain lignin models. The Δδ values of the A 2, C 2^(I)^, B 5, C 5^(I)^, C 5^(II)^, A 6, C 6^(I)^, and C 6^(II)^ signals obtained from the aromatic regions of the long-chain lignin models were all larger than 0.006 ppm on the ^1^H axis, whereas the Δδ values of the A 2 and C 5^(I)^ signals on the ^13^C axis were greater than 0.05 ppm (Fig. 5a). For the short-chain lignin models, only the Δδ values of the B 5 signals on the ^1^H axis exceeded 0.006 ppm (Fig. 5b). Line broadening was observed in the B 5 signals of the long-chain lignin models in the presence of *Tr*CBM1 (350 μM). The observed line broadening could be attributed to both the on and off rates of the complex formation and multiple binding states of lignin due to nonspecific binding [22].

### Adsorption experiments with the lignin models

The unlabeled long- and short-chain lignin models of compound **4** were used to evaluate *Tr*CBM1 binding affinity based on the Langmuir adsorption model. The *M*ws and molecular weight distributions of the unlabeled lignin models (Table 1) were nearly identical to those of the ^13^C-labeled lignin models (Additional file 1: Figure S2). The *Tr*CBM1–His tag fusion protein was used instead of *Tr*CBM1 without a His tag to conduct the adsorption experiments because the soluble lignin models could not be separated from the *Tr*CBM1 protein via centrifugation. After incubating the sample solutions containing the lignin models and *Tr*CBM1–His tag at 50 °C for 1 h, cOmplete His-Tag Purification Resin (Roche, Basel, Switzerland) was added to the solutions. The unbound lignin models in the supernatant could then be separated from the bound lignin models, which were adsorbed on the precipitated *Tr*CBM1–His tag that was bound to the His tag resin. By performing control experiments, we confirmed that all of the *Tr*CBM1–His tag could adsorb onto the His tag resin. No undesired binding was observed between the His tag resin and lignin models (Additional file 1: Figures S6 and S7). To calculate the adsorption parameters summarized in Table 2, the concentrations of the unbound lignin models in the supernatants were determined. Although the long- and short-chain lignin models showed similar amount of the lignin model bound to His tag–*Tr*CBM1 at saturation (Γ_max_), the Langmuir affinity constant (K_L_) of the long-chain lignin model was eight times higher than that of the short-chain lignin model. This result was consistent with that of the NMR interaction analysis. The percentages of the lignin models that bound to *Tr*CBM1 were calculated using the K_L_ values. In the CSP analysis, 84.6% and 91.4% of the long-chain lignin models were bound to *Tr*CBM1 at *Tr*CBM1 concentrations of 200 and 350 μM, respectively. At *Tr*CBM1 concentrations of 200 and 350 μM, 33.0% and 46.6% of the short-chain lignin models, respectively, were bound to *Tr*CBM1. The chain length was, thus, an essential factor in the binding between *Tr*CBM1 and the lignin chains, which exclusively contained β-*O*-4 linkages. We also found that a DP of greater than 4 was required for significant adsorption.

**Table 2:** *Tr*CBM1–His tag adsorption parameters of the unlabeled lignin models based on the Langmuir adsorption model

Lignin model	Langmuir affinity constant, K_L_ (mL/mg)	Amount of adsorption at saturation, Γ_max_ (μg/mg)
Long chain	36.26	8.77
Short chain	4.47	9.15

## Discussion

Understanding the interaction mechanism between cellulase and lignin is essential for efficient enzymatic saccharification of lignocelluloses. *T. reesei* is the most important industrial cellulase-producing filamentous fungus. Cel7A is the most abundant secreted cellulolytic enzyme and contains a catalytic domain and *Tr*CBM1. The nonproductive binding between *Tr*CBM1 and lignin decreases the efficiency of enzymatic saccharification of pretreated plant biomass; hence, the interaction mechanism has been previously studied. The flat plane surface of the *Tr*CBM1 binds to both lignin and cellulose and contains three tyrosine residues (Y5, Y31, and Y32) and neighboring amino acid residues in the underside of *Tr*CBM1 (Fig. 1) [9, 27, 28]. T17, V18, and T24 residues are present in the cleft, which is located on the opposite side of the flat plane surface. This site also interacts with lignin and cellulosic substrates [22]. Although the *Tr*CBM1 binding sites have been extensively investigated using the point mutation analysis and NMR, the evidence to identify the lignin atoms participating in *Tr*CBM1 binding is lacking. Therefore, we conducted the CSP analysis to investigate the interactions between ^13^C-labeled β-*O*-4 lignin oligomer compounds and *Tr*CBM1.

Our CSP results clearly indicated that the long-chain lignin model showed a stronger affinity for *Tr*CBM1 than the short-chain lignin model (Table 2). Mattinen et al. found that cellohexaose adsorbed on the *Tr*CBM1, whereas short cellooligosaccharides, such as cellobiose and cellotriose, did not bind to *Tr*CBM1 [29]. Based on these results and our observations, stacking on the flat plane surface of *Tr*CBM1 and interactions with the cleft required lignin and cellooligosaccharides with long chains. The low affinity of the short-chain lignin model could also be attributed to its conformation. The short-chain lignin model adopted a folded conformation in 90% water [25], and the folded short lignin chain could not cover the full length of the three tyrosine residues on the flat plane surface of *Tr*CBM1. Therefore, we surmise that the β-*O*-4 lignin chains with DPs above 4 were a prerequisite for the strong adsorption of *Tr*CBM1.

**Fig. 6:**
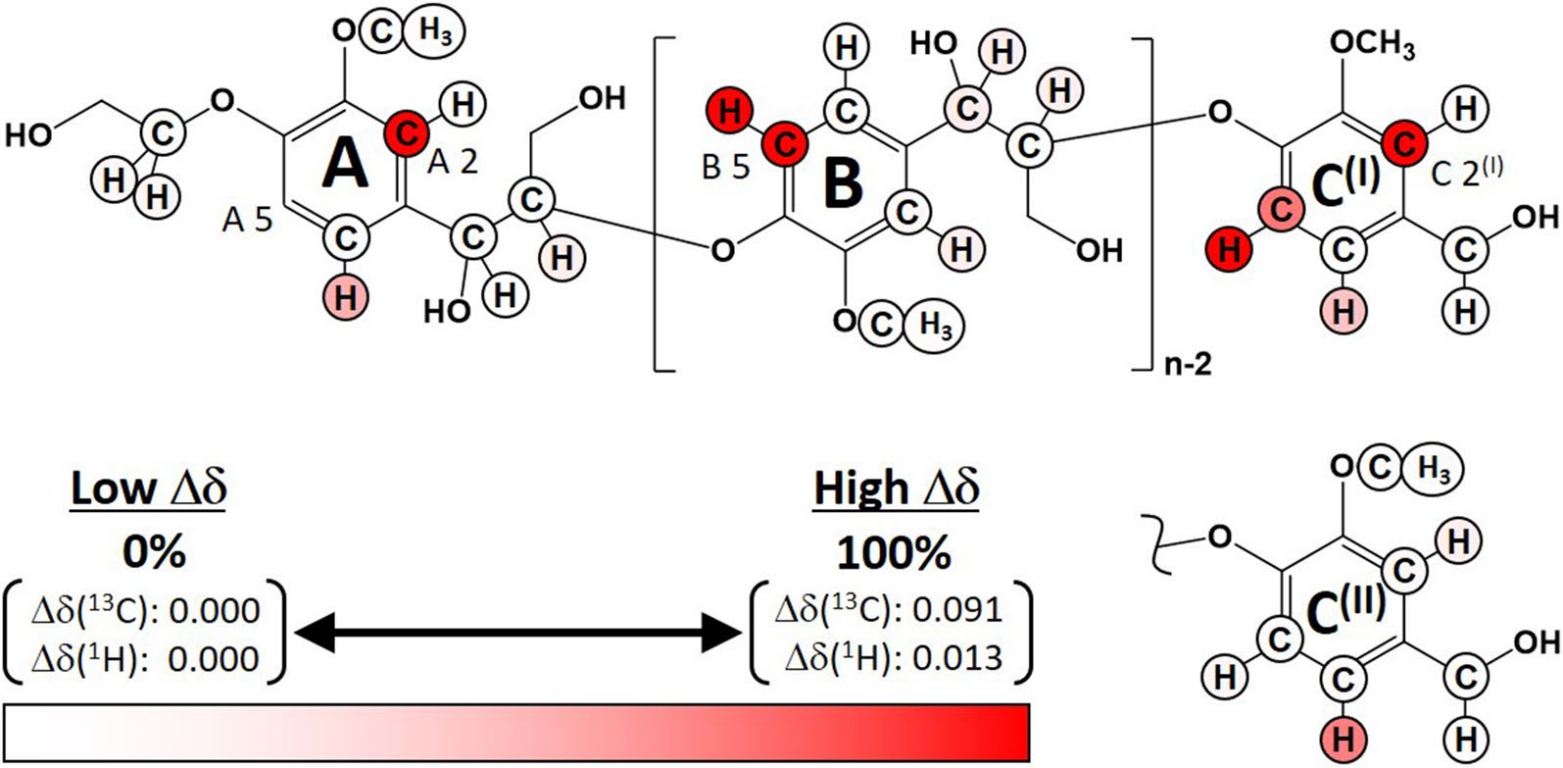
Mapping of lignin model binding sites with *Tr*CBM1. The Δδs observed in the CSP experiment using the long-chain lignin model with 350 μM of *Tr*CBM1 are shown by red gradation, which corresponds to the relative intensities of Δδs. Differently aligned terminal units of two different configurations, C^(I)^ and C^(II)^, are exhibited. In the A 2 and C 2^(I)^ positions, the Δδ value is the same because their 2D HSQC signals were not distinguishable due to overlapping

The interactive sites in the long-chain lignin model are mapped in Fig. 6; these sites are based on the Δδ values observed in the CSP analysis. The interactions between the aromatic rings and *Tr*CBM1 were obvious, whereas the aliphatic regions and methoxy groups did not exhibit major binding sites. This finding suggests that the adsorption of *Tr*CBM1 on lignin occurs via hydrophobic interactions and π–π stacking of the aromatic rings in lignin and three tyrosine residues on *Tr*CBM1. Rahikainen et al. discussed the importance of *Tr*CBM1 hydrophobicity. They reported that a Y32A *Tr*CBM1 mutant exhibited a lower association constant than the wild-type *Tr*CBM1, whereas a Y32W mutant increased the lignin and cellulose binding affinities of *Tr*CBM1 [9]. The hydrophobicity of lignin causes a significant amount of nonproductive binding with cellulase [17, 30]. Therefore, the hydrophobic interaction can reasonably be interpreted as a dominant driving force behind nonproductive *Tr*CBM1 binding by lignin.

Interestingly, a variety of Δδ in the aromatic rings of the lignin model is observed (Fig. 6). The terminal units on the A and C^(I)^ rings exhibited similar interaction patterns, although the A 5 position was not evaluated due to its extremely weak HSQC signal intensity. The internal units, B ring, showed remarkable interactions with *Tr*CBM1 only at the B 5 position, suggesting that the interaction patterns of the terminal and internal units of the lignin model differed. We previously reported two configurations of the lignin models whose terminal units were differently aligned and designated them C^(I)^ and C^(II)^ [25]. We demonstrated that the *Tr*CBM1 binding behavior significantly depended on the lignin model configurations. The molecular alignment of C^(I)^ apparently resulted in preferential binding to *Tr*CBM1. Thus, we are the first to reveal that the interactive sites in the lignin chains are significantly influenced by their molecular configurations.

Hydrogen bonding and electrostatic interactions are not negligible in nonproductive *Tr*CBM1 binding. The phenolic OH groups in lignin promote hydrogen bonding to cellulolytic enzymes [14, 31]. The hydrophilic amino acid residues in *Tr*CBM1, including Q7 and T17 in addition to the main chain of H4 and I11, were previously shown to participate in binding with lignin via hydrogen bonds and electrostatic interactions [22]. In our CSP analysis, aromatic rings in the lignin models were the primary sites for interactions with *Tr*CBM1. This was mainly attributed to the hydrophobic interactions. However, the results could also be interpreted in terms of hydrogen–π interactions between the aromatic rings in lignin and OH groups in the amino acid residues [32, 33]. The OH groups in the three tyrosine residues on the flat plane surface of *Tr*CBM1 and those in the T17 residue in the cleft were assumed to donate hydrogen for the hydrogen–π interactions. Although the CSP analysis indicated that the interactions at the aliphatic positions were insignificant compared with those at the aromatic positions, the signals from the α positions exhibited slightly higher Δδ values than those from other aliphatic positions in the lignin models (Fig. 5). This result could be attributed to hydrogen binding between OH groups near the α positions of the lignin model and *Tr*CBM1.

## Conclusions

Highly efficient enzymatic saccharification is hindered by the nonproductive binding of lignin to cellulolytic enzymes. Until now, the binding mechanism was not fully understood. We synthesized ^13^C-labeled β-*O*-4 lignin oligomer model compounds to identify the *Tr*CBM1 binding sites in lignin using the NMR CSP analysis. The signals from the aromatic regions in the lignin models exhibited obvious perturbations, whereas those from the aliphatic regions and methoxy groups were insignificantly perturbed. These results suggested that the aromatic rings in the lignin models are major sites for interactions with *Tr*CBM1. *Tr*CBM1 bound differently with the terminal and internal units in the lignin models. Moreover, the binding patterns associated with the C^(I)^ and C^(II)^ terminal alignments differed, indicating that the binding of the lignin models to *Tr*CBM1 was strongly affected by the molecular configuration. The perturbation of the signals of the long-chain lignin models (DP 4.16–4.70) was obvious because of their strong binding affinity relative to that of the short-chain lignin models (DP 2.64–3.12). This finding indicated that a chain length greater than DP 4 was necessary for strong interactions between lignin and *Tr*CBM1. In the complex between *Tr*CBM1 and the long-chain lignin model, the aromatic rings of the lignin are core interactive sites for hydrophobic interactions and π–π stacking with *Tr*CBM1. A detailed understanding of the nonproductive binding will facilitate the establishment of a fundamental theory for the structural alteration of lignin and enzymes that are unsusceptible to unfavorable binding.

## Methods

### Materials

*E. coli* BL21 (DE3) was obtained from Merck (Darmstadt, Germany). Vanillins with the ^13^C-labeled carbonyl group and aromatic ring were obtained from Cambridge Isotope Laboratories (Tewksbury, MA, USA). Vanillin with the ^13^C-labeled methoxy group and [2-^13^C]-*t*-butyl-2-bromoacetate were purchased from Taiyo Nippon Sanso (Tokyo, Japan). Other laboratory chemicals were obtained from Wako Pure Chemical Industries (Osaka, Japan), nacalai tesque (Kyoto, Japan), and Tokyo Chemical Industry (Tokyo, Japan).

### Preparation of *Tr*CBM1

The CBM1 of *T. reesei* Cel7A (accession number: CAH10320) was expressed and purified as described previously [22]. *E. coli* BL21 (DE3) was first subjected to heat shock transformation with plasmids for the expression of His-tagged *Tr*CBM1 fused with GFP, hereafter referred to as His tag–*Tr*CBM1–GFP fusion protein. The LB medium was inoculated with the transformant, followed by incubation at 37 °C and 200 rpm until the OD_600_ reached 1.2. Further, protein expression was induced using 1 mM isopropyl β-1-thiogalactopyranoside, and the bacteria were incubated at 37 °C and 200 rpm for 5 h. Following centrifugation and sonication, the His tag–*Tr*CBM1–GFP was isolated from the supernatant and purified by Ni affinity chromatography followed by anion exchange chromatography. The His tag and GFP regions were removed using enterokinase (New England Bio Labs, MA, USA) and thrombin (GE Healthcare, IL, USA), respectively. The obtained *Tr*CBM1 was concentrated in 50 mM acetic acid-*d*_4_ buffer prepared with D_2_O using a Vivaspin turbo ultrafiltration device (Sartorius, Göttingen, Germany). We skipped the His tag cleavage process to prepare His tag–*Tr*CBM1 for the adsorption analysis. The His tag–*Tr*CBM1 was purified by Ni affinity and anion exchange chromatography.

### Synthesis of β-*O*-4 lignin oligomer model compounds

The β-*O*-4 lignin oligomer models were synthesized as described in our previous work using a modified protocol originally developed by Katahira et al. [25, 34]. Vanillin (**1**) was dissolved in acetone, and the solution was refluxed for 1.5 h in the presence of KI, K_2_CO_3_, and *t*-butyl-2-bromoacetate to obtain *t*-butoxycarbonylmethyl vanillin (**2**). The monomer (**2**) was dissolved in anhydrous THF, and 1.5 M LDA was added dropwise to the solution under nitrogen atmosphere over 30 min at a constant temperature of −30 °C. The solution was stirred for 30 min at −30 °C and for an additional 1.5 h at 0 °C–5 °C to obtain the polymerized oligomer (**3**). Oligomer **3** was dissolved in *t*-butanol containing NaBH_4_. Methanol was added to the mixture stepwise over 1 h under constant stirring at 65 °C–70 °C, and the mixture was stirred for 6 h to obtain β-*O*-4 lignin oligomer model compound **4**. The synthesized lignin models were separated into long- and short-chain fractions by silica gel chromatography. Sequential elution was performed using (1) ethyl acetate, (2) ethyl acetate/methanol (5:1, v/v), and (3) ethyl acetate/methanol (2:1, v/v). Three types of the ^13^C-labeled lignin model compounds were synthesized for the CSP analysis. Vanillins with ^13^C-labeled aromatic rings (**1**^(Ar)^) and [2-^13^C]-*t*-butyl-2-bromoacetate were used as starting materials to synthesize a lignin model with ^13^C-labeled aromatic rings and β positions (**4**^(Arβ)^). Similarly, vanillins with ^13^C-labeled carbonyl and methoxy groups were used to synthesize a lignin model with ^13^C-labeled α positions (**4**^(α)^) and a model with ^13^C-labeled methoxy groups (**4**^(m)^). The lignin models were characterized using 2D ^1^H–^13^C HSQC NMR and SEC. SEC was performed using three TSKgel SuperMultipore HZ-M columns (Tosho, Tokyo, Japan) on a Shimadzu instrument equipped with an LC-20AD pump and SPD-M20A diode array detector (Shimadzu, Kyoto, Japan). THF was used for elution at a flow rate of 0.35 mL/min at 40 °C.

### NMR spectroscopy and CSP analysis

All NMR spectra were recorded at 298 K on a Bruker Avance III HD 600 spectrometer equipped with a cryogenic probe and Z gradient (Bruker BioSpin, MA, USA). The instrument was controlled using Bruker Topspin NMR software (version 3.5). For the CSP analysis, 50 μM of the ^13^C-labeled lignin models with the ^13^C-labeled aromatic rings and β positions (**4**^(Arβ)^), α positions (**4**^(α)^), and methoxy groups (**4**^(m)^) were individually dissolved in 50 mM acetic acid-*d*_4_ buffer, which was prepared using D_2_O (pD 5.0) and 10% (v/v) DMSO-*d*_6_. Each NMR sample comprised a volume of 250 μL in a 5-mm Shigemi symmetrical microtube (Shigemi, Tokyo, Japan) and contained 20 μM 2,2-dimethyl-2-silapentane-5-sulfonic acid as an internal standard. To identify the *Tr*CBM1 binding sites in the ^13^C-labeled lignin models, changes in the chemical shift (Δδ_C,H_, ppm) were calculated by comparing the chemical shift values in the edited ^1^H–^13^C HSQC spectra of the ^13^C-labeled lignin models in the presence and absence of 200 and 350 μM *Tr*CBM1. The CSP analysis of both the long- and short-chain lignin models was performed to evaluate the effect of molecular weight on *Tr*CBM1 binding to the lignin models. The assignment of the lignin model signals was based on our previous report [25]. All NMR experiments were performed in 90% D_2_O because enzymatic saccharification is typically conducted in aqueous media, and the solubility of the lignin models was good in the NMR solvent owing to their low molecular weights.

### Adsorption experiment of lignin models with *Tr*CBM1

The Langmuir adsorption isotherm model was used to evaluate the *Tr*CBM1 binding affinities of the lignin models. Each unlabeled long- and short-chain lignin model was dissolved in 50 mM acetic acid buffer (pH 5.0) containing 3,200 μg/mL of *Tr*CBM1 with a His tag (His tag–*Tr*CBM1). The lignin models were prepared in 1.5-mL microtubes at concentrations of 10, 20, 30, 50, 80, 100, 150, and 200 μM. The total volume of each solution was 50 μL. The sample solutions were incubated at 50 °C for 60 min under constant shaking at 1,000 rpm using a Comfort thermomixer (Eppendorf, Hamburg, Germany). To each solution, 100 μL of a 50% (v/v) suspension of complete His-Tag Purification Resin (Roche, Basel, Switzerland) in the 50 mM acetic acid buffer was added. The mixtures were resuspended by vortexing for 10 s and centrifuged at 500*g* for 10 s. The concentrations of the free lignin model in the supernatants were determined by measuring the absorbance at 280 nm using a BioSpec-nano spectrophotometer (Shimadzu, Kyoto, Japan). The quantity of each lignin model bound to *Tr*CBM1 was calculated by subtracting the amount of free lignin model compound from the initially loaded amount. Experiments were conducted in duplicate, and the average values were used to calculate the Langmuir affinity constants using Eq. (1)

1 $$ \varGamma_{C} \,\text{ = }\,\varGamma_{\hbox{max} } \,\left[ {K_{L} \,C\text{/}\,\left( {1\,\text{ + }\,K_{L} \,C} \right)} \right], $$

where Γ_C_ is the amount of the bound lignin model, Γ_max_ is the amount of the lignin model bound to His tag–*Tr*CBM1 at saturation, K_L_ is the Langmuir affinity constant, and C is the concentration of the free lignin model in the supernatant. A solution containing only His tag–*Tr*CBM1 without a lignin model was used as a blank.

## Supplementary information

**Additional file 1: Figure S1.** *Tr*CBM1 purity analysis using SDS-PAGE and MALDI-TOF-MS. **Figure S2**. Molecular weight distributions of the ^13^C-labeled and unlabeled β-*O*-4 lignin oligomer model compounds. **Figure S3.** 2D ^1^H–^13^C HSQC spectra of the short-chain ^13^C-labeled lignin oligomer models in the CSP experiment. **Figure S4.** Full 2D ^1^H–^13^C HSQC spectra from the CSP experiments. **Figure S5.** Full 2D ^1^H–^13^C HSQC spectrum of 350 μM *Tr*CBM1. **Figure S6.** UV–vis spectra of the supernatant of the *Tr*CBM1–His tag control used in the adsorption experiments. **Figure S7.** UV–vis spectra of the supernatants obtained in the adsorption experiments with the long- and short-chain models.

## Data Availability

All data generated or analyzed during this study are included in this manuscript and Additional files.

## Abbreviations

CSP: Chemical shift perturbation
DMSO: Dimethyl sulfoxide
DP: Degree of polymerization
EXSY: Exchange spectroscopy
G: Guaiacyl
GFP: Green fluorescent protein
His tag: Histidine tag
HMBC: Heteronuclear multiple bond correlation
HSQC: Heteronuclear single-quantum correlation
LDA: Lithium diisopropylamide
LR-HSQMBC: Long-range heteronuclear single-quantum multiple bond correlation
MALDI-TOF-MS: Matrix-assisted laser desorption/ionization time-of-flight mass spectrometry
*M*w: Weight-average molecular weight
MWL: Milled wood lignin
NMR: Nuclear magnetic resonance
ROESY: Rotating-frame Overhauser effect spectroscopy
S: Syringyl
SDS-PAGE: Sodium dodecyl sulfate-polyacrylamide gel electrophoresis
SEC: Size exclusion chromatography
THF: Tetrahydrofuran
TOCSY: Total correlation spectroscopy
