# Supplementary material for: NMR elucidation of nonproductive binding sites of lignin models with carbohydrate-binding module of cellobiohydrolase I
Source: Biotechnol Biofuels. 2020 Oct 7;13:164. doi: 10.1186/s13068-020-01805-w (PMC7541279; doi:10.1186/s13068-020-01805-w)
Supplement: Supplementary file 1 — Additional file 1: Figure S1. TrCBM1 purity analysis using SDS-PAGE and MALDI-TOF-MS. Figure S2. Molecular weight distributions of the 13C-labeled and unlabeled β-O-4 lignin oligomer model compounds. Figure S3. 2D 1H–13C HSQC spectra of the short-chain 13C-labeled lignin oligomer models in the CSP experiment. Figure S4. Full 2D 1H–13C HSQC spectra from the CSP experiments. Figure S5. Full 2D 1H–13C HSQC spectrum of 350 μM TrCBM1. Figure S6. UV–vis spectra of the supernatant of the TrCBM1–His tag control used in the adsorption experiments. Figure S7. UV–vis spectra of the supernatants obtained in the adsorption experiments with the long- and short-chain models. [file 13068_2020_1805_MOESM1_ESM.docx]

Additional file

**NMR Elucidation of Nonproductive Binding Sites of Lignin Models with Carbohydrate-Binding Module of Cellobiohydrolase I**

Yuki Tokunaga^1^, Takashi Nagata^2^, Keiko Kondo^2^, Masato Katahira^2^, and Takashi Watanabe^1^*^,^

*Correspondence: twatanab@rish.kyoto-u.ac.jp

^1^ Research Institute for Sustainable Humanosphere (RISH), Kyoto University, 611-0011, Uji, Kyoto, Japan

^2^ Institute of Advanced Energy (IAE), Kyoto University,611-0011, Uji, Kyoto, Japan

1. Characterization of unlabeled *Tr*CBM1

The molecular mass of *Tr*CBM1 was determined using SDS-PAGE. The purity of *Tr*CBM1 was determined via MALDI-TOF-MS analysis using an Autoflex III instrument (Bruker Daltonics, MA, USA). *Tr*CBM1 was detected as a single band in the SDS-PAGE gel. The MALDI-TOF-MS spectrum contained a single molecular ion peak at *m*/*z* 5,196.8 ([M+H]^+^).

**
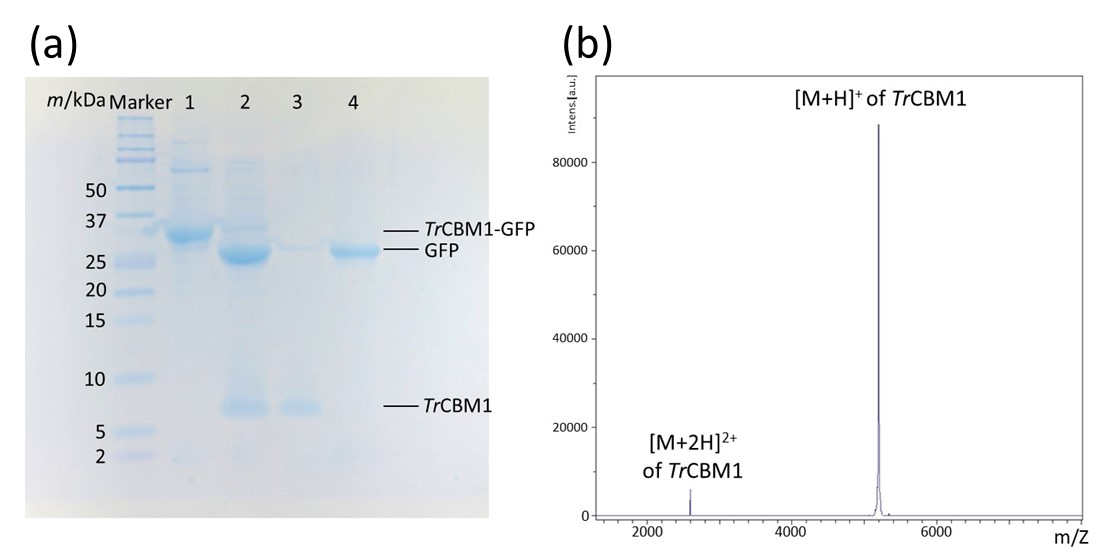
**

**Figure S1. *Tr*CBM1 purity analysis.** (a) SDS-PAGE results for *Tr*CBM1 obtained at each stage of purification. Lanes 1 and 2 show the protein fractions obtained before and after GFP cleavage by thrombin. Lanes 3 and 4 show *Tr*CBM1 and GFP after passing through and trapping on a benzamidine column, respectively. (b) MALDI-TOF-MS spectrum of *Tr*CBM1.

2. Molecular weight distributions of the lignin models

The molecular weights of the lignin models were determined via SEC under the conditions described in the experimental section. The molecular weight parameters are summarized in Table 1 in the main text.


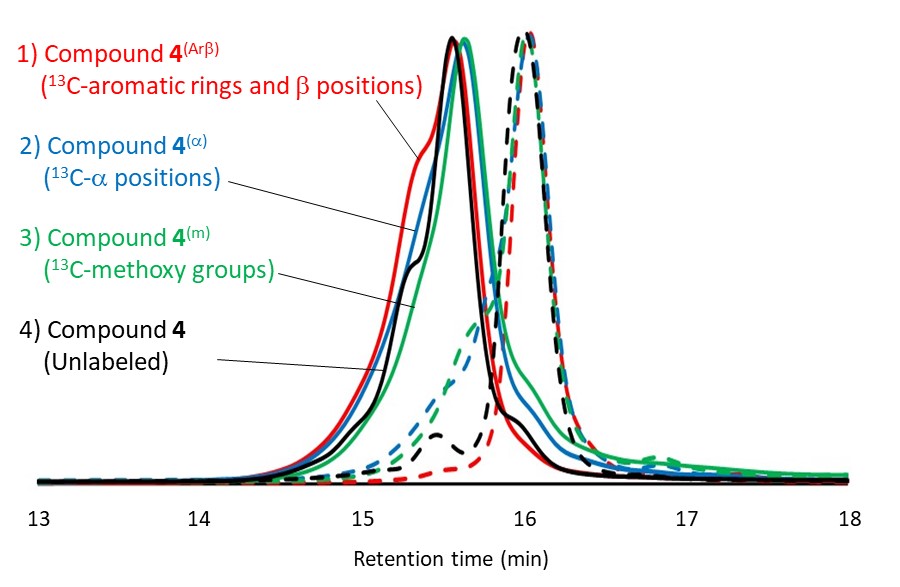


**Figure S2. Molecular weight distributions of ^13^C-labeled and unlabeled β-*O*-4 lignin oligomer model compounds.** GPC profiles of the ^13^C-labeled lignin models are indicated in red (**4**^(Arβ)^), blue (**4**^(α)^), and green (**4**^(m)^). Profiles of the unlabeled lignin models are indicated in black. The profiles of the long chains are represented by solid lines, and those of the short chains are shown as dotted lines.

3. CSP analysis of short-chain lignin models


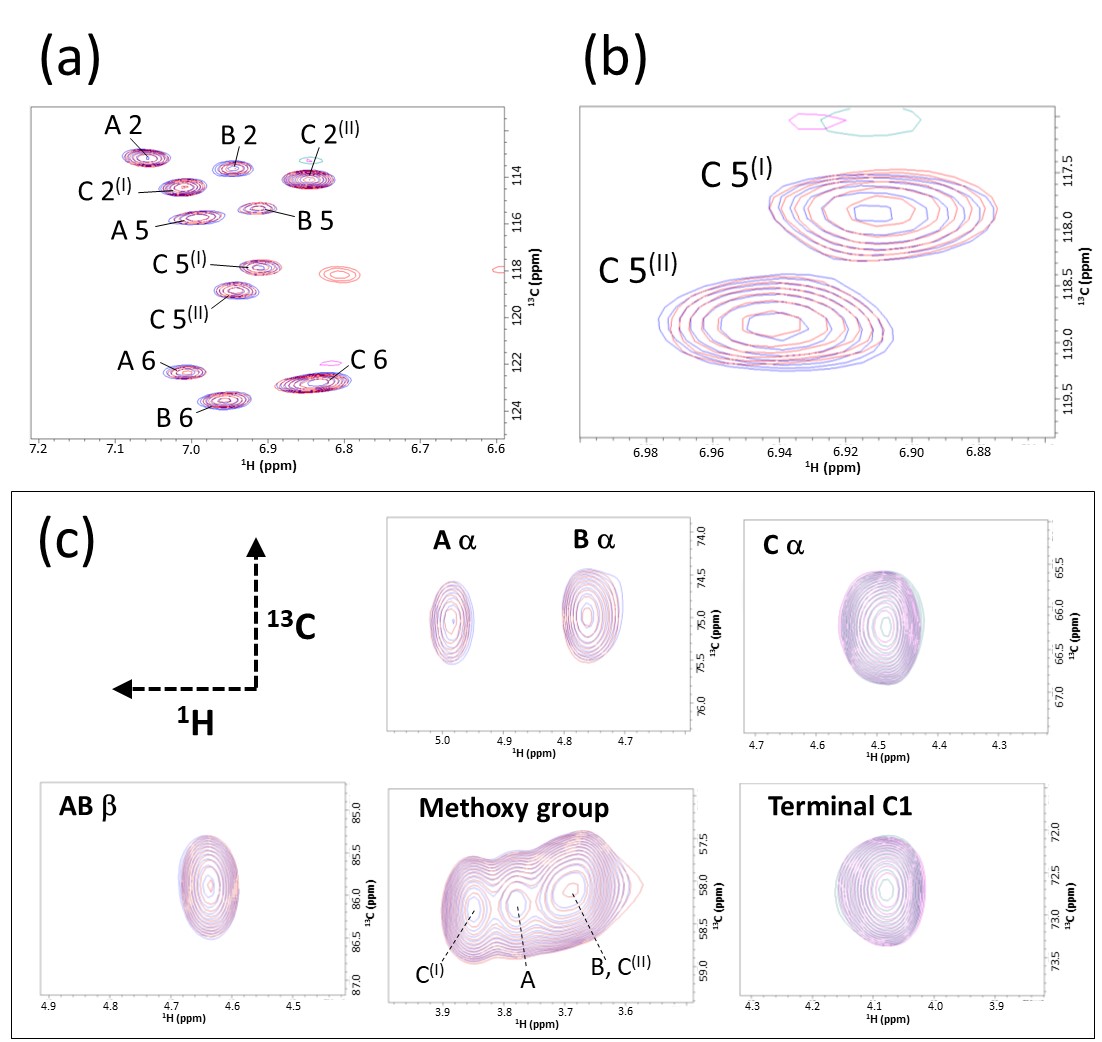


**Figure S3. 2D ^1^H-^13^C HSQC spectra of the short-chain ^13^C-labeled lignin oligomer models in the CSP experiment.** (a) Superimposed 2D ^1^H-^13^C HSQC spectra of the aromatic region in compound **4**^(Arβ)^ (50 μM) in the presence (red) and absence (blue) of 350 μM *Tr*CBM1. (b) A magnified image of the HSQC signals from the C 5 positions. (c) Superimposed 2D ^1^H-^13^C HSQC spectra of aliphatic regions and methoxy group in **4**^(Arβ)^, **4**^(α)^, and **4**^(m)^ (50 μM) in the presence (red) and absence (blue) of 350 μM *Tr*CBM1.


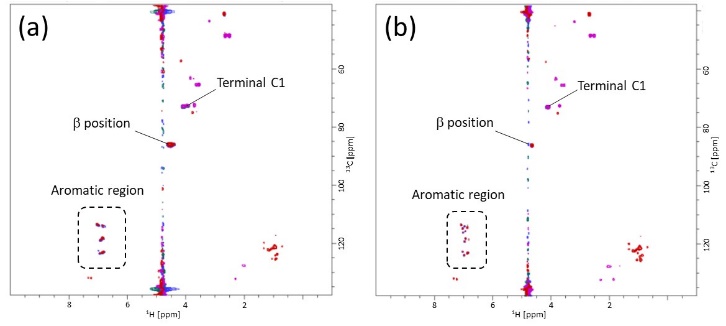
4. Full spectra from the CSP experiment


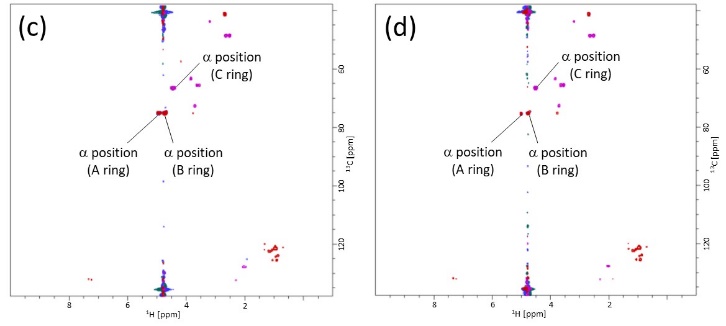


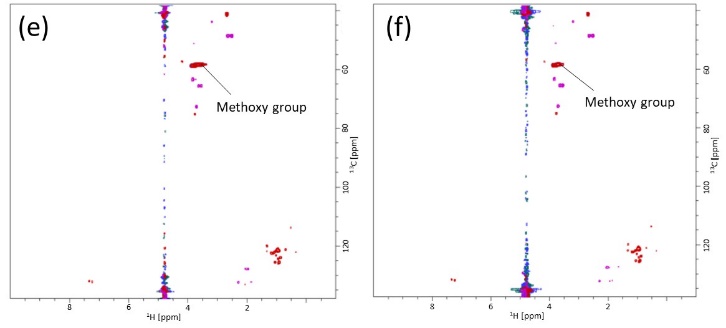


**Figure S4.** **Full 2D ^1^H-^13^C HSQC spectra from the CSP experiments.** The CSP analysis was performed using the ^13^C-labeled lignin models and *Tr*CBM1 in 50 mM acetic acid-*d*_4_ buffer prepared with D_2_O (pD 5.0) and 10% (v/v) DMSO-*d*_6_. 2D ^1^H-^13^C HSQC spectra of the lignin model with (a and b) ^13^C-labeled aromatic rings and β positions (**4**^(Arβ)^), (c and d) ^13^C-labeled α positions (**4**^(α)^), and (e and f) ^13^C-labeled methoxy groups (**4**^(m)^). The HSQC spectra of the long-chain lignin models are shown in panels a, c, and e (left). The spectra of the short-chain lignin models are shown in panels b, d, and f (right). The HSQC signals in the spectra of the ^13^C-labeled lignin oligomer models in the presence (red) and absence (blue) of 350 μM *Tr*CBM1 are superimposed.

**
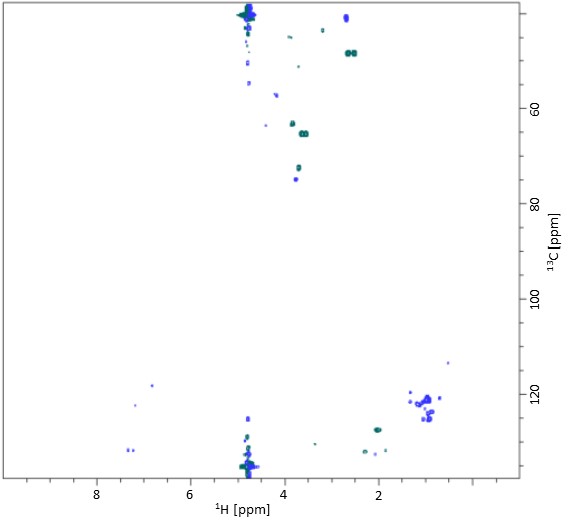
**

**Figure S5. Full 2D ^1^H-^13^C HSQC spectrum of 350 μM *Tr*CBM1**. The HSQC spectrum of *Tr*CBM1 in 50 mM acetic acid-*d*_4_ buffer prepared using D_2_O (pH 5.0) and 10% (v/v) DMSO-*d*_6_.

5. Control experiments for the adsorption analysis

*Tr*CBM1–His tag was used as a control in the adsorption experiment. After the addition of His tag resin and centrifugation, absorbance by the supernatant at 280 nm decreased to 0.7% of the initial value (Fig. S6). This result indicated that the *Tr*CBM1–His tag was completely bound to the His tag resin. Compound **4** was used as the lignin model in the adsorption experiment. After the addition of His tag resin and centrifugation, the absorbances of the supernatants of the long- and short-chain lignin model solutions at 280 nm were 32.7% and 34.4% of the initial values (Fig. S7). The volumes of the sample solutions had increased to 150 μL from initial volumes of 50 μL after adding the His tag resin. The results indicated that the lignin models remained in the supernatants without binding to the His tag resin. This enabled us to analyze the *Tr*CBM1 binding affinities of the lignin models with a high degree of accuracy.

**
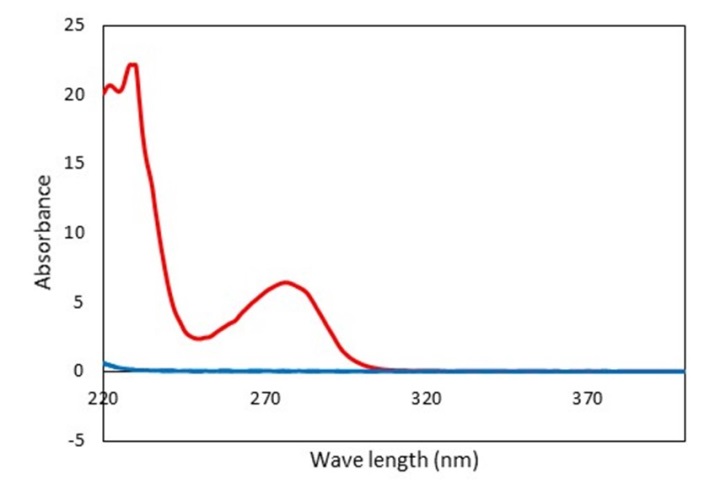
**

**
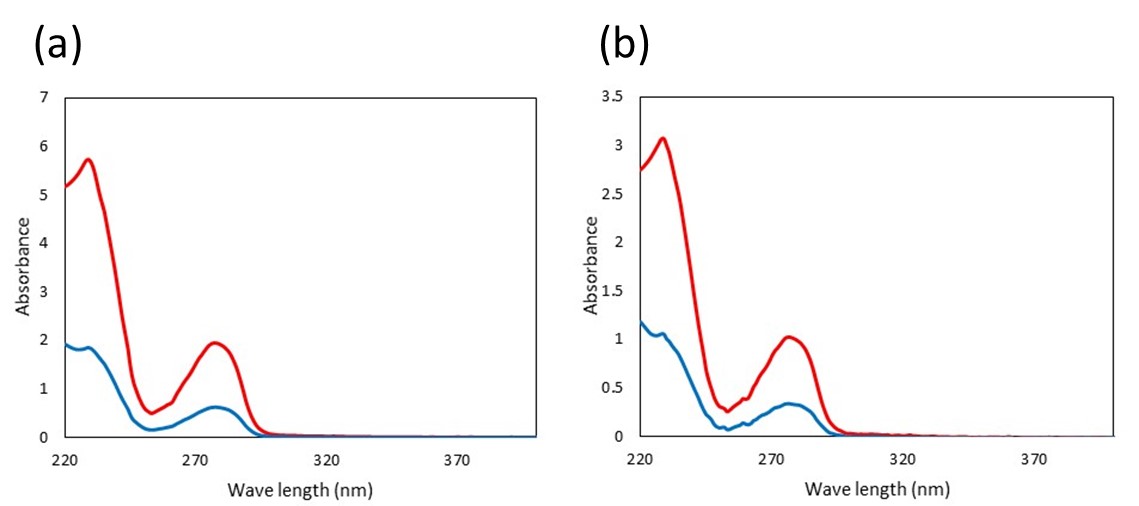
Figure S6. UV-vis spectra of supernatant of the *Tr*CBM1–His tag control used in the adsorption experiments.** The red line is the UV-vis spectrum of the initial supernatant containing the *Tr*CBM1–His tag. The blue line is the UV-vis spectrum of the supernatant after addition of the His tag resin and subsequent centrifugation.

**Figure S7.** **UV-vis spectra of supernatants obtained in adsorption experiments with (a) long-chain and (b) short-chain models.** The red lines are the UV-vis spectra of the initial supernatants containing the lignin models. The blue lines are the UV-vis spectra of the supernatants after the addition of His tag resin and subsequent centrifugation.
